# Literary Retrospect

**Published:** 1863-01

**Authors:** 


### LITERARY RETROSPECT.

*Clinical Medicine.—Observations recorded at the Bedside, with Commentaries.* By W. T. GAIRDNER, Physician to the Royal Infirmary of Edinburgh, and Lecturer on the Practice of Medicine. (Edmonston and Douglas.) 8vo, pp. 741. 1862.

THE present work of Dr. Gairdner is essentially of the Edinburgh model of a medical book. As we have perused its pages the days of Cullen have returned on us, Home has flitted across the field, and ghosts even of Brunonians, demanding a place for a ghostly moment, have been chased off by Alison, that he might himself have their place. It is strange how thoughts, works, and tendencies cling to these old haunts of science: you may know anywhere a thoroughbred Edinburgh physician at once by hearing him or reading him. They are all clinical, even in the most laborious sense of that term. The bedside is their world: not a thing in that sphere of theirs that is not noted. They could build a substantial hospital out of their very note-books. It seems as though nothing in the way of symptoms of disease could possibly escape them—escape their eyes, their ears, their touch, their smells, and we had almost said their taste; for we have known them taste with evident satisfaction things which the majority of the world would put aside—nay, record the event in the note-book, "diagnosis from the specific gravity, confirmed by the sweet and honey-like taste."

The Edinburgh physicians are consequently men to be profoundly admired; they earn admiration and get it paid them. They invariably get large practices wherever they go; their secret of success, their intense attention to detail. Your accomplished English physician, entering the sick room, remarks on the weather, says a word, perhaps, about Parliament, or if he sees a picture or work of art, introduces his professional conversation with an expression of opinion as to the author of the work. If he does this with consummate skill he now and then wins golden prizes; but on the broad scale, he is nowhere compared with your thorough Edinburgh at-it-at-once clinicaller; whose "Show me your tongue, sir," is followed instanter by the warning, "Nurse, I should like, before I go, just to see anything that may have passed from the booels."

There are, unquestionably, great benefits to be derived from the study of clinical medicine, but there are disadvantages also. The benefits lie in the spirit of accuracy that is engendered, the care, the natural familiarity with disease; the disadvantages in the narrowness of the sphere over which the intellect ranges, in the tendencies, which grow with the growth and increase with the strength, to believe that there is nothing to be considered in medical science but bedside observation; to ignore the great outer universe; and to detach symptoms from their external and ruling causes.

Dr. Gairdner's volume, built on the pure clinical model, is a world of labour. He gives us eighteen chapters or sections, and an appendix. He opens with a retrospect of cases treated in his infirmary during the session 1855-56; he passes forwards to treat on the subjects of pneumonia, the use of alcoholics, the duty of a physician respecting the same, influenza, typhus and enteric fever, epidemic fever of Edinburgh, scarlatina, cholera, syphilis, hysteria, delirium tremens, dipsomania, pleuritic effusion, pneumothorax, miscellaneous cases of pulmonary diseases, aneurism, and cardiac murmurs; he furnishes a retrospect of 200 cases under treatment during the winter session of 1859-60, adds sundry hints on the study of clinical medicine, and ends with an appendix.

It would be unjust to Dr. Gairdner did we not say that, considering the character of the work in hand, he has made it as interesting as is well possible; he has put his observations in the form of lectures, has illustrated them freely with numerous effective sketches, and has given a clearness and precision to the different topics which have been discussed that has long been needed.

To learn diagnosis in cases such as have been described, to collect facts, to ensure acquaintance with some new diagnostic truth in regard to every subject discussed, the student and practitioner may consult Dr. Gairdner with advantage. They will find in him a man who is always at home and has something useful to say.

To attempt to analyse the work would be a failure; it is not a book for analysis nor even for special criticism, inasmuch as there is no point for disputation in it anywhere; and although it erects its own tomb, it has a certain measure of life that will bear a good many burials, and turning up again, in course of time, as fresh as ever. It is, in a sentence, a true Edinburgh picture, drawn from nature with pre-Raphaelite fidelity, of a large class of diseases which are not very likely to leave mankind within the age of letters, and which will be persistent in letters so long as *Gairdner's Clinical Medicine* finds place in Dryasdust's library.

---

*Chemistry.* By William Thomas Brande, D.C.L., F.R.S.L. & E., and Alfred Swaine Taylor, M.D., F.R.S. (J. W. Davies.) Small 8vo, pp. 892.

Yet another to the many works on Chemistry adapted for the tyro. But whatever the number of manuals on, or introductions to, this subject, whatever the plethora of the market with regard to it, an elementary treatise from the pens of two of our most veteran teachers and greatest authorities on chemistry, compels a welcome reception. There is a novelty in this book of Drs. Brande and Taylor which will doubtless secure for it a principal place among text-books for chemical students. It assumes, in fact, the form of a species of protest of Old Chemistry against the style of teaching in vogue with Young Chemistry. The former avows that the latter, intoxicated, as as it were, by the great advances of chemical science, has been going a-head too fast, and attempting too much in the indoctrination of the science, to the risk of bewildering hopelessly the student. Drs. Brande and Taylor tell us that, "without departing from the true objects of Chemistry, its facts admit of explanation in a form intelligible to any educated man. It is not necessary to the progress of this Science (they maintain), that its language should change with the opinions of every new theorist. The numerical value of atoms and volumes is not of so much importance to a student, as a correct description of the properties and uses of the substances which they represent. On this part of the subject much labour appears to us to have been wasted by certain writers. They have apparently been engaged in working out an idea, and seeking for some Utopian standard of perfection, in a new system of notation: but in endeavouring to settle contested points on a firmer basis, they have incurred the risk of unsettling everything. Thus, instead of pursuing the inductive method, and fitting hypotheses to facts, they have introduced a deductive system, by which facts are made to bend to hypotheses; and the elementary composition of bodies is altered, in order that they may correspond to certain artificial types. There is an old French proverb which it may be well to bear in mind, in reference to this practice: "Quand la Nature dit que telle chose est, et l'homme dit que telle chose n'est pas, il faut en croire la Nature."

Without attempting to enter into the lists with such formidable antagonists, it may, however, be very fairly questioned whether the assertion is not too broad that "a correct description of the properties and uses of the substances which they represent" is of chief importance to the chemical student. With the majority of students, particularly medical students, neither a very accurate nor very intimate acquaintance with chemistry is, as a rule, possible. It is necessary that what they do learn should be accurately learned, but it is of infinitely more importance, as a question of mental cultivation and practical utility, that they should be indoctrinated in the *spirit* rather than in the facts of chemistry. An individual might know every fact in chemistry, and yet not be a true chemist. With the medical student, at least, chemistry is an acquirement necessary to be pursued only so far as to enable him the better to comprehend and to master other acquirements. If he be taught the mere concrete facts of the science, he is taught chiefly what is its immediate practical bearing, and what is most certainly known. He is taught the alphabet and the simplest combinations of its letters; but he is left in ignorance of those complex combinations, and potential properties of the letters, without which they are in a great degree dead letters. The highest teachings and greatest powers of chemistry are included in those discussions on the numerical value of atoms and volumes, which Drs. Brande and Taylor seem so positively to condemn. In the broad fashion in which they have put their objections to a system of teaching which is based upon theoretical considerations, these objections would almost seem to come into the category of those "dangerous errors," which, as Sir W. Hamilton says, (Lect. i. p. 5,) "consist in regarding the cultivation of our faculties as subordinate to the acquisition of knowledge, instead of regarding the possession of knowledge as subordinate to the cultivation of our faculties."

Our authors continue :—"While we do not undervalue the usefulness of a correct language to express the facts and theories of a science, we have the strongest objection to frequent and capricious changes, in which the good is uncertain and prospective while the evil is certain and immediate." In the present work, then, they have adopted that "simple chemical language which has found acceptance in the schools and colleges of Great Britain, France, and Germany, as well as in the best treatises on the science." They have not professed to write a treatise, but to provide the student and general reader with a plain introduction to the subject. The book constitutes a Manual of Practical Chemistry, including Practical Toxicology (so far as the most important poisons are concerned), and Photography. With regard to the latter art, the authors have, as fully as their space would permit, treated of the chemical principles on which it is based, and have given some practical rules for the guidance of those who wish to apply their chemical knowledge to this interesting pursuit. Pictorial illustrations have been excluded from the work, on the ground that "those who require this aid to their studies, will find in the illustrated catalogues of dealers in chemicals more correct representations of apparatus, than those which are commonly met with in treatises on chemistry."

Of the execution of this work, as conceived by the authors, it is idle to speak. Their names alone are a sufficient guarantee for its completeness.

*Travels in Peru and India, while superintending the Collection of Chinchona Plants and Seeds in South America, and their Introduction into India.* By Clements R. Markham, F.S.A., F.R.G.S. (Murray.) 8vo, pp. 572. 1862.

For some time back anxiety has been felt respecting the future supplies of quinine-yielding cinchona, or rather, as Mr. Markham instructs us to spell the word, *chinchona.* It was the name of the Countess of *Chinchon*, not Cinchon, which was given by Linnæus to the genus of trees yielding the famous *bark.* From the entire absence of all attempts at conservation in the great South American forests from which we derive Chinchona bark, the supplies are becoming restricted, and threaten to become precarious sooner or later; and as a consequence the high price of quinine seriously diminishes the use which might be made of that invaluable drug.

A notion of the importance of the questions involved in the maintenance of our supplies of Chinchona bark may be gathered from the following isolated figures. Previous to 1857 the Government of India expended 7000*l.* a year in quinine. In 1857 the expenditure rose to 12,000*l.*, but at the present time the cost of the quinine and bark used in the Government hospitals very largely exceeds the last-named sum, and quinine is retailed by the government druggists at 1*l.* an ounce. A writer in the *Friend of India* (Dec. 10, 1860) estimates the expenditure for quinine and bark in the Government hospitals in India for the two years 1857-8 at 54,520*l.*; and for 1858-9, at 40,696*l.*!

The commercially valuable species of Chinchona are as follows:—

| | | |
|---|---|---|
| | C. succiruba | yielding *Red bark.* |
| C. Condaminea. | C. Chahuarguera | *Crown bark.* |
| C. Condaminea. | C. crispa | *Crown bark.* |
| C. Condaminea. | C. Uritusinga | *Crown bark.* |
| C. Condaminea. | C. lancifolia | *Carthagena bark.* |
| | C. nitida | *Grey bark.* |
| | C. micrantha | *Grey bark.* |
| | C. Peruviana | *Grey bark.* |
| | C. Calisaya | *Yellow bark.* |

These different kinds of medicinal bark are collected from five different regions in South America: to wit, (1) the Loxa region, notable for its *crown barks;* (2) the *red bark* region on the western slopes of Chimborazo; (3) the New Grenada region; (4) the Huanuco region in Northern Peru, notable for *grey barks;* and (5) the Calisaya region in Bolivia and Southern Peru.

The collection of the different barks has been pursued in the most reckless manner. A century ago Condamine raised a warning voice against the destruction then going on in the forests of Loxa. Humboldt reported that 2,500 trees were destroyed every year. The readiest mode is commonly taken by the collectors for obtaining the bark—the tree being cut down without reference to subsequent growth. Although all that is required for the preservation of vitality in the stump, is to cut the stem as near as possible to the root, even this simple precaution is rarely attended to. As a consequence, in certain districts valuable species of chinchona have been almost extirpated; while in others the bark has to be sought further and further within the recesses of the forest.

In 1839 Dr. Royle, in his work on Himalayan botany, advocated the introduction of quinine-yielding trees into India; and in 1852, he submitted an able memorandum on the subject to the Indian Government, pointing out that "the home supply of a drug which already costs 7000*l.* a year, would be advantageous in an economical point of view, and invaluable as affording means of employing a drug which is indispensable in the treatment of Indian fevers. I have no hesitation in saying (he adds) that, after the Chinese teas, no more important plant could be introduced into India." No effectual steps were taken to carry out this important suggestion until 1859, when Mr. Markham was instructed to make such arrangements as would best ensure the success of attempts to procure quinine-yielding plants in Peru, and their safe conveyance to India. Mr. Markham was eminently fitted for this task, from a previous knowledge of the district to be explored, and of the native tongue. In what manner he carried his instructions into effect, how he overcame the local trade jealousy and opposition to his mission, and how happily he succeeded, are all told in this most interesting and valuable work. All the commercially valuable species of chinchona have been introduced into India, and are now rapidly growing there; and there is the fairest promise that the bark of this plant will become one of the most important commercial products of the Indian Empire, as well as an invaluable boon to the natives of that vast territory. On the 31st August, 1862, 72,568 healthy plants were growing on the Neilgherry Hills alone, 13,700 of which had been permanently placed in the plantations prepared for them.

*Air and Water; their Impurities and Purification.* By HENRY HOLLMAN CONDY. (J. W. Davies.) 8vo., pp. 78. 1862.

In this work Mr. Condy treats of the disinfecting action of the Alkaline Permanganates—a subject with which his name has become inseparably connected. Those who wish to familiarize themselves with this subject, and with the properties of *Condy's Disinfecting Fluid*, will do well to refer to this work.

*Wisdom, Intelligence, and Science, the True Characteristics of Emanuel Swedenborg. With special reference to a recent Article in the Medical Critic and Psychological Journal.* By MEDICUS CANTABRIGIENSIS. (J. S. Hodson and Son.) 8vo. 1862.

We would willingly have passed this pamphlet unnoticed, but for a remark towards the close which requires a word of explanation on our part. The author characterizes a statement made by the writer of the article on "*Swedenborg's Dreams*" in this Journal (vol. i. p. 612) as a "gross inaccuracy." The statement is to the effect that the writer believed that English, French, and German translations of the *Dreams* exist, but that the publication of the English translation (prepared by the Swedenborg Society) has been suppressed; and he intimates the probability, from the impossibility of procuring copies, that the French and German translations had also been suppressed. Medicus Cantabrigiensis writes: "We are in a position to deny, from positive knowledge, that the Swedenborg Society prepared or published any translation whatever of these Dreams. We can also vouch for the fact that Swedenborg's friends have had nothing to do with the suppression of any English translation of the work; nor do we believe that there has been any suppression at all by any other party." Curiously enough, the Swedish editors of the "*Dreams*," as quoted by Medicus Cantabrigiensis, state that—"This remarkable document was originally not intended for general circulation, and has, therefore, been communicated only to some few persons of lofty sentiments who are interested in the subject. Since the multiplication of translations into English, French, and German, no reason can be found for denying to the original a greater publicity in Swedenborg's native country. Hence a literal copy of Herr Kleming's edition is now, with his own sanction, rendered accessible to the book trade." Respecting the statement of the "multiplication of translations," in the languages referred to, Medicus Cantabrigiensis writes, in a foot-note, "This appears to be *a mistake.* We cannot learn that these translations exist." It is manifest that a writer who characterises what he believes to be a like error, in one case as a "gross inaccuracy," and in another as simply "a mistake," betrays an animus which it is needless to do more than point out.

All the information which the writer of the article criticized possesses, on an English translation of the "*Dreams*," was derived from the manager or agent of the Swedenborg Society, in Bloomsbury-street, soon after the first announcement of the publication of the "*Dreams*," on the Continent, by the Berlin correspondent of the *Daily Telegraph.* The agent not only stated that an English translation of the "*Dreams*" had been prepared, but that several copies of this translation were in existence, and he offered to procure the writer of the Article the loan of a copy. Subsequently he told the writer, that it had been determined not to publish the English translation of the "*Dreams*." If the writer erred, from the source of his information, in believing that the Swedenborg Society was the active body in causing the "*Dreams*" to be translated, and the translation to be suppressed, the error was at least as pardonable as that which the Swedish editors of the "*Dreams*" have committed.

When Medicus Cantabrigiensis appeals to the publication of the "*Dreams*" in Sweden, as testimony against all desire of concealment on the part of Swedenborg's admirers, and further implies that the writer of the Article was guilty of suppression in not mentioning this fact, he forgets that the writer had manifested more respect for the editors of the "*Dreams*" than he himself does. The writer gave the editors the credit of publishing the "*Dreams*" simply on account of their "great value in enabling us to judge of Swedenborg's psychical condition" at the time when the dreams occurred: Medicus Cantabrigiensis is careful to point out that the editors did not intend the original document for general circulation, and did not give it such circulation until privacy was no longer possible.

Medicus Cantabrigiensis states of Swedenborg's "Memorable Relations," that "it is clear that they must be either so many hallucinations, or so many wilful falsehoods, or, as we do not hesitate to call them, so many sublime truths; in one or other of these three categories they must be placed; in which of them, let truth and equity decide." Admitting that these opposing conclusions may be derived from these "Memorable Relations," he takes umbrage that the writer of the Article adopts the least obnoxious of the two which he himself rejects. He, however, although a physician, makes no attempt to show in what manner the facts cited by the writer from the "*Dreams*" are misinterpreted, but simply asks, whether it is common justice, to test Swedenborg's mental sanity, not by the articles which he wrote, but by his private and occasional record of his dreams, "intended for no other eye than his own."

Medicus Cantabrigiensis forgets that the Swedish editors of the "*Dreams*" write of this diary, that "exhibiting the transition period of Swedenborg's life, the change from the worldly to the spiritual, these notes are of great value in enabling us to judge of his psychical condition, which they represent to us as extremely agitated, and into which we can thus look more deeply than was before possible." If the writer of the Article has been unjust, the admirers of Swedenborg have been more so; but to give the argument of Medicus Cantabrigiensis any weight, it would be necessary to show that no faith ought to be placed in the facts recorded in the "*Dreams*."

Medicus Cantabrigiensis entirely avoids the true question raised in the Article against which he protests, namely, the medico-psychological signification of the facts recorded in the "*Dreams*;" at times he adopts a phraseology seriously at variance with literary cour tesy, and for which no justification can be found in the Article upon which he animadverts.

*Practical Notes on Diagnosis, Prognosis, and Treatment in Cases of Delirium Tremens.* By THOMAS LAYCOCK, M.D., F.R.S.E., &c., Professor of the Practice of Medicine, &c., in the University of Edinburgh. Edin. 8vo, pp. 27. 1862.

We earnestly commend this reprint on the advantages to be derived from the expectant and rational treatment of delirium tremens to our readers. The argument is conclusive; but for the right application at the bedside of the conclusions flowing from it, accuracy of diagnosis and prognosis are needed, to both of which this pamphlet will furnish important aid. One or two remarks on the nomenclature of delirium tremens may be cited here with advantage:—

"It is to be observed, *in limine*, that both in nosology and in practice, the term delirium tremens applies to a group of disorders, whereas, strictly and literally taken, it designates but one of the group. By it we thus understand an acute cerebral affection caused by intoxicating drinks, of which delirium and tremors are the prominent symptoms. Other acute cerebral affections are accompanied or manifested by delirium and tremors, but then they are differentiated from delirium tremens by the causation. Strictly, however, we ought not to include other acute cerebral affections caused by the habitual use of intoxicating drinks, which are not manifested by delirium and tremors, under the same term; but, then, in excluding these, we lose a practical advantage, for they resemble the typical form in all essential points. They have the same origin, run the same course, and are generally amenable to the same treatment. They are, in short, of the same genus, though not the same species. A more serious hindrance to diagnosis and prognosis is presented in the fact that the acute cerebral disorder is not always caused by intoxicating drinks: these rather modify its symptoms and course. This is specially observable in the primary stages of certain forms of insanity, when there is more or less of oinomanïa. How are these mixed forms to be classed? . . . .

"The use of a name is simply that the thing named may be made known in speech and writing. Hence the practical character of the nomenclature in general, for it names the groups of symptoms so definitely caused as those comprised under the term delirium tremens, by the name of the cause. And this is the more necessary because the same groups of symptoms are often otherwise caused. This object is best gained, however, by adding an adjective like "methystic," as expressive of the cause, to the name of any nervous disorder caused by drunkenness; and, thus, both the origin and cause are indicated, which such a term as delirium does not express; while at the same time thereby we differentiate it from the disorder when otherwise caused, and in this way indicate both the cause and the leading symptoms. This would fail, however, in regard to the classification of those cases which result from other nervines than fermented and distilled drinks; for it is, I think, well established, that the excessive use of opium or its salts, of tobacco, and even of certain bitters usually thought harmless tonics, will excite, though more rarely, the same class of affections; while in other countries, other drugs besides opium and tobacco are used abusively with similar consequences. The word 'phrenesia,' adopted by Albers, would be a good term to indicate the entire group of acute mental disorders caused by the abuse of nervine stimuli in general, and 'methystic' might be used adjectively to indicate those genera which are caused either directly or predisponently by drunkenness. The phrases '*neuropathia* potatorum,' and 'alcoholismus nervorum chronicus,' include all diseases of the nervous system caused by drunkenness, and are therefore inapplicable here.* What, then, are the cerebral affections—duration and cause apart—which are grouped under the term delirium tremens when caused by intoxicating drinks? They are known as hypochondriasis; as melancholia in its various forms,—apprehensive, suspecting, aggressive, suicidal; as insane impulses—to kill, burn, or otherwise destroy, drink to excess (oinomania), and gratify insanely the appetites and instincts; as illusions, hallucinations, delusions, and delusive apprehensions; as wakeful and trembling delirium; and, finally, as mania. These may pass into each other, or be complicated with each other, as when melancholia passes into mania, or insane impulses to gratify instincts and appetites are complicated with hallucinations: or they may be complicated with motor neuroses, as tremors, spasmodic jerkings, convulsive fits, epilepsy; and finally may end either in death or become chronic, when the affection comes to be classed as some form of insanity. Any case of this kind, with these leading characters, as to cause, duration, symptoms, and treatment, might be designated methystic phrenesia or phrenesy; and each particular form, methystic hypochondriasis, methystic melancholia, methystic delirium, and the like, according to the predominant mode of mental disturbance. (pp. 12—14.)

*A Year Book of Medicine, Surgery, and their Allied Sciences, for* 1861. Edited by Dr. Harley, Dr. Handfield Jones, Mr. Hulke, Dr. Grailey Hewitt, and Dr. Sanderson, for the New Sydenham Society. 1862.

In commenting upon the previous volume of the *Year Book*, we remarked that it was "a decided advance, both in arrangement and compilation, upon its predecessor, and gave promise of a still better future." The *Year Book* for 1861 is a decided advance upon that for 1860.

On our table we have also lying *Researches and Observations on*

---

* See Falck in *Virchow's Handbuch der Speciellen Pathologie und Therapie*, bd. ii. p. 303.

*Pelvic Hæmatocele*, by J. Byrne, M.D., Resident Fellow of the New York Academy of Medicine, &c. (New York.) Mr. Yearsley's *Introduction to the Art of Laryngoscopy*, reprinted from the *Medical Circular;* an Essay *On the Causes of the Evils incident to Infant Dentition*, by J. C. Clendon, M.R.C.S.; and *An Appeal to Physiologists and the Press*, by H. Freke, A.B., M.B.

